# Pembrolizumab plus axitinib versus sunitinib in metastatic renal cell carcinoma: outcomes of Japanese patients enrolled in the randomized, phase III, open-label KEYNOTE-426 study

**DOI:** 10.1007/s10147-021-02014-7

**Published:** 2021-11-20

**Authors:** Satoshi Tamada, Chihiro Kondoh, Nobuaki Matsubara, Ryuichi Mizuno, Go Kimura, Satoshi Anai, Yoshihiko Tomita, Masafumi Oyama, Naoya Masumori, Takahiro Kojima, Hiroaki Matsumoto, Mei Chen, Mengran Li, Kenji Matsuda, Yoshinobu Tanaka, Brian I. Rini, Hirotsugu Uemura

**Affiliations:** 1 Bell Land General Hospital, Higashiyama 500-3, Naka-ku, Sakai, Osaka 599-8247 Japan; 2 Toranomon Hospital, 2 Chome-2-2 Toranomon, Minato City, Tokyo, 105-8470 Japan; 3 National Cancer Center Hospital East, 6 Chome Kashiwanoha, Kashiwa, Chiba 277-0882 Japan; 4 Keio University Hospital, Shinanomachi, Shinjuku City, Tokyo, 〒160-8582 Japan; 5 Nippon Medical School Hospital, 1-1-5 Sendagi Bunkyo, Tokyo, 113-8603 Japan; 6 Nara Medical University Hospital, 840 Shijo-cho, Kashihara, Nara 634-8522 Japan; 7 Niigata University Medical & Dental Hospital, 1-757 Asahimachi, Chuou-ku, Niigata, 951-8510 Japan; 8 Saitama Medical University International Medical Center, 1397-1 Yamane, Hidaka, Saitama 350-1298 Japan; 9 Sapporo Medical University, S1, W16, Chuo-ku, Sapporo, 060-8543 Japan; 10 University of Tsukuba, 1 Chome-1-1 Tennodai, Tsukuba, Ibaraki 305-8577 Japan; 11 Aichi Cancer Center, 1-1 Kanokoden, Chikusa-ku, Nagoya 464-8681, Japan; 12 Yamaguchi University, 1-1-1, Minami-Kogushi, Ube, Yamaguchi 755-8505 Japan; 13 Merck & Co., Inc., 2000 Galloping Hill Road, Kenilworth, NJ 07033 USA; 14 MSD K.K., Kitanomaru Square, 1 Chome-13-12 Kudankita, Chiyoda City, Tokyo, 102-0073 Japan; 15 Cleveland Clinic Taussig Cancer Institute, CA Building, 10201 Carnegie Ave, Cleveland, OH 44106 USA; 16 Present Address: Vanderbilt-Ingram Cancer Center, 1301 Medical Center Dr #1710, Nashville, TN 37232 USA; 17 Kindai University, 3 Chome-4-1 Kowakae, Higashiosaka, Osaka 577-8502 Japan

**Keywords:** Metastatic renal cell carcinoma, Axitinib, PD-1 checkpoint inhibitor, Pembrolizumab, Vascular endothelial growth factor receptor inhibitor

## Abstract

**Background:**

In the phase III open-label KEYNOTE-426 (NCT02853331) study, first-line pembrolizumab and axitinib improved overall survival (OS) and progression-free survival (PFS) versus sunitinib for metastatic renal cell carcinoma (mRCC). KEYNOTE-426 evaluated patients enrolled from 25 sites in Japan.

**Methods:**

Patients enrolled in Japan were included in this post hoc subgroup analysis. Adults with clear cell mRCC were randomly assigned 1:1 to receive intravenous pembrolizumab 200 mg every 3 weeks plus oral axitinib 5 mg twice daily or oral sunitinib 50 mg once daily (4 weeks on/2 weeks off). Dual primary endpoints were OS and PFS as assessed by blinded independent central review. Objective response rate (ORR) and safety were secondary endpoints.

**Results:**

The Japanese subgroup comprised 94 patients (pembrolizumab–axitinib, *n* = 44; sunitinib, *n* = 50; 11% of the intent-to-treat population). Median time from randomization to data cutoff (January 6, 2020) was 29.5 months (range 24.6–37.3). Consistent with the intent-to-treat population, the OS, PFS, and ORR suggested improvement with pembrolizumab–axitinib versus sunitinib in the Japanese subgroup. Grade ≥ 3 treatment-related adverse events (TRAEs) occurred in 70% of patients receiving pembrolizumab–axitinib versus 78% receiving sunitinib; 11 (25%) patients receiving pembrolizumab–axitinib and 13 (27%) patients receiving sunitinib discontinued the study medication due to AEs. TRAEs led to the discontinuation of pembrolizumab, axitinib, pembrolizumab–axitinib, or sunitinib in 32%, 34%, 14%, and 20%, respectively. No deaths from TRAEs occurred.

**Conclusions:**

Efficacy outcomes for the Japanese subgroup were consistent with those of the global population. Safety in Japanese patients was consistent with the results from the global population.

**Supplementary Information:**

The online version contains supplementary material available at 10.1007/s10147-021-02014-7.

## Introduction

Approximately 24,000 people in Japan were diagnosed with kidney cancer in 2018, with about 8000 deaths [1]. Renal cell carcinoma (RCC) accounts for 90% of kidney cancers [2]. In Japan, the recommended first-line treatments for metastatic RCC (mRCC) are pembrolizumab with axitinib or avelumab with axitinib for patients regardless of International Metastatic Renal Cell Carcinoma Database Consortium (IMDC) risk group, sunitinib or pazopanib for patients with favorable or intermediate IMDC risk, and ipilimumab with nivolumab or cabozantinib for patients with intermediate or poor IMDC risk [3]. Real-world analyses have found that nearly half of Japanese patients with mRCC receiving standard-of-care first-line therapy had died before receiving second-line therapy (follow-up 22 months) [4], indicating a high level of unmet need for first-line therapies with improved efficacy.

Programmed death 1 (PD-1) and its ligand PD-L1 are highly expressed in the tumor microenvironment of mRCC based on a study using samples from Japanese patients [5] and from the Cancer Genome Atlas [6], indicating that PD-1/PD-L1 inhibitors may be effective in this cancer type. Pembrolizumab is a monoclonal antibody that binds to the PD-1 receptor and blocks its interaction with PD-L1 and PD-L2 [7]. Pembrolizumab monotherapy demonstrated promising antitumor activity in treatment-naive patients with either clear cell (cohort A) [8] or nonclear cell RCC (cohort B) [9] in the phase II, single-arm, open-label, multicohort KEYNOTE-427 trial. The objective response rate (ORR) was 36.4% (*N* = 110) and 26.7% (*N* = 165) in cohorts A and B, respectively. At the first interim analysis of the randomized, open-label, phase III KEYNOTE-426 trial (NCT02853331) [10], patients with treatment-naive mRCC given the combination of pembrolizumab plus axitinib (pembrolizumab–axitinib) versus sunitinib alone demonstrated superior overall survival (OS) and progression-free survival (PFS), and a greater proportion of patients achieved an objective response. Pembrolizumab–axitinib has now become a standard-of-care therapy [10, 11]. After additional follow-up, pembrolizumab–axitinib demonstrated sustained improvements in efficacy compared with sunitinib [12]. Pembrolizumab–axitinib is approved as first-line therapy for advanced or mRCC in the United States [7], the European Union [13], Japan [3], and other countries/regions.

Here we present the efficacy and safety outcomes of pembrolizumab–axitinib versus sunitinib for the subset of patients enrolled in the KEYNOTE-426 study in Japan.

## Patients and methods

### Study design and patient population

Details on the KEYNOTE-426 study design and results of the first interim [10] and extended follow-up [12] analyses have been published previously. Briefly, adult patients with newly diagnosed stage IV or recurrent mRCC with clear cell histology, with no prior systemic treatment for advanced disease, with measurable disease according to Response Evaluation Criteria in Solid Tumors, version 1.1 (RECIST v1.1), and a Karnofsky Performance Status (KPS) scale score of ≥ 70 at baseline were enrolled from 129 sites across 16 countries. The present post hoc subgroup analysis included patients enrolled at 25 sites in Japan. This study was conducted in accordance with Good Clinical Practice Guidelines and the Declaration of Helsinki, and the study protocol was approved by the institutional review board or ethics committee of each participating site [12]. All patients provided written informed consent to participate before enrollment.

### Treatment and assessments

Enrolled patients were stratified by IMDC risk categories (favorable vs. intermediate or poor) and geographic regions (North America vs. Western Europe vs. Rest of the World) and then randomized in a 1:1 ratio to pembrolizumab 200 mg intravenously every 3 weeks plus axitinib 5 mg orally twice daily (pembrolizumab–axitinib group) or sunitinib 50 mg orally once daily for 4 weeks then off treatment for 2 weeks (sunitinib group). The treatment continued for 35 cycles until progressive disease, unacceptable toxicity, or study withdrawal. Patients treated with pembrolizumab–axitinib could continue one drug if the other was discontinued for toxicity. Imaging was performed at week 12, then every 6 weeks through week 54, and then every 12 weeks; response assessments used RECIST v1.1. Survival status was assessed every 12 weeks, and adverse events (AEs) were recorded through 30 days after the last dose of trial treatment (90 days for serious AEs). Subsequent therapies were permitted after study treatment discontinuation according to investigator discretion.

### Study end points

The dual primary endpoints of KEYNOTE-426 were OS (time from randomization to any-cause death) and PFS (time from randomization to progression or any-cause death). Secondary endpoints were ORR (proportion of patients who have complete or partial response [CR or PR]) and duration of response (DOR) (time from first documented evidence of response to progression or any-cause death). PFS, ORR, and DOR were assessed according to RECIST v1.1 by blinded independent central review. Safety and tolerability were secondary endpoints. The National Cancer Institute Common Terminology Criteria for Adverse Events version 4.0 was used for AE reporting. Immune-mediated AEs were reported based on a list of terms specified by the sponsor, could be associated with drug exposure, and could be consistent with an immune phenomenon representing an immunological etiology.

### Statistical analyses

All efficacy endpoints were analyzed using the intention-to-treat population (all randomly assigned patients, analyzed in the group to which they were randomized). Safety was assessed using data from the as-treated population (all randomly assigned patients who received ≥ 1 dose of study treatment).

Details of statistical analyses of KEYNOTE-426 and results of the first interim analysis and extended follow-up were reported previously [10, 12]. The primary and key secondary endpoints were met at the time of the first interim analysis. The median time from randomization to death or the date of data cutoff was 12.8 months for the first interim analysis (randomization to data cutoff: 14.2 months) [10]. The median time from randomization to the date of data cutoff was 30.6 months for the extended follow-up [12].

PFS, OS, and DOR were analyzed using the Kaplan–Meier method. Hazard ratios (HRs) and 95% confidence intervals (CIs) for the OS and PFS endpoints were estimated using the unstratified Cox regression model with the Efron method of tie handling with treatment as a covariate.

## Results

### Patients

**Fig. 1:**
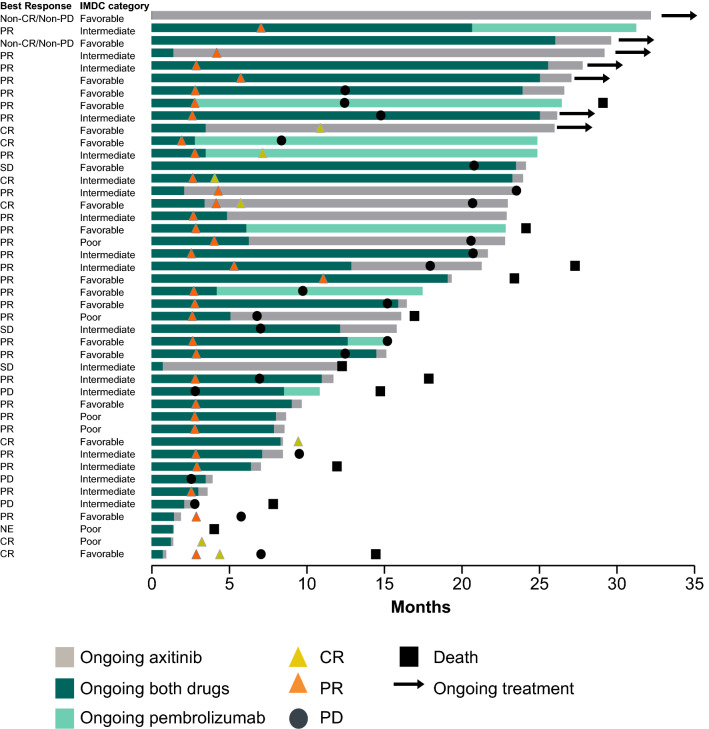
Response over time in the Japanese population in the pembrolizumab–axitinib group (*n* = 44). Pembrolizumab was administered for a maximum number of 35 cycles (actual doses), with 21 days per cycle (± 3 days), and some patients may have missed dose cycles; pembrolizumab treatment may therefore extend past 2 years in some cases. *CR* complete response, *IMDC* International Metastatic Renal Cell Carcinoma Database Consortium, *PD* progressive disease, *PR* partial response, *SD* stable disease

A total of 861 patients were randomized (pembrolizumab–axitinib: 432, sunitinib: 429), of whom 94 (11%) were enrolled in Japan (pembrolizumab–axitinib: 44, sunitinib: 50). Median follow-up time from randomization to data cutoff (January 6, 2020) was 29.5 months (range 24.6–37.3) in the Japanese population. At this data cutoff, 33 of 44 (75%) patients in the pembrolizumab–axitinib group and 42 of 49 (86%) in the sunitinib group who received ≥ 1 dose of study drug had discontinued treatment. Most of the discontinuations in both treatment groups were due to disease progression (pembrolizumab–axitinib: *n* = 17 [39%]; sunitinib: *n* = 24 [49%]) or AEs (pembrolizumab–axitinib: *n* = 11 [25%]; sunitinib: *n* = 13 [27%]) (Supplementary Fig. 1). Four patients (9%) completed the full 2 years of pembrolizumab. The treatment was ongoing in 7 patients in the pembrolizumab–axitinib group (16%) and 7 (14%) patients in the sunitinib group (Fig. 1 and Supplementary Fig. 1).

Baseline characteristics of the Japanese subgroup were generally consistent with the global population, although the Japanese subgroup did include older patients, more patients with favorable IMDC risk, and a larger proportion of patients with KPS scale scores of 90/100 versus 70/80 (Supplementary Table 1). A total of 24 (73%) and 39 (93%) patients in the pembrolizumab–axitinib and sunitinib groups, respectively, received subsequent therapy after discontinuation of study drug; 7 (21%) and 25 (60%) patients, respectively, received ≥ 2 subsequent lines (Supplementary Table 2). The most commonly used subsequent therapies in the pembrolizumab–axitinib arm were pazopanib in 9 (27%), axitinib in 6 (18%), and sunitinib in 5 (15%) patients. In the sunitinib arm, the most commonly used subsequent therapies were nivolumab in 28 (67%), axitinib in 18 (43%), and pazopanib in 6 (14%) patients. A vascular endothelial growth factor (VEGF)/VEGF receptor (VEGFR) inhibitor was used in 18 (55%) and 26 (62%) patients, respectively.

### Efficacy

**Fig. 2:**
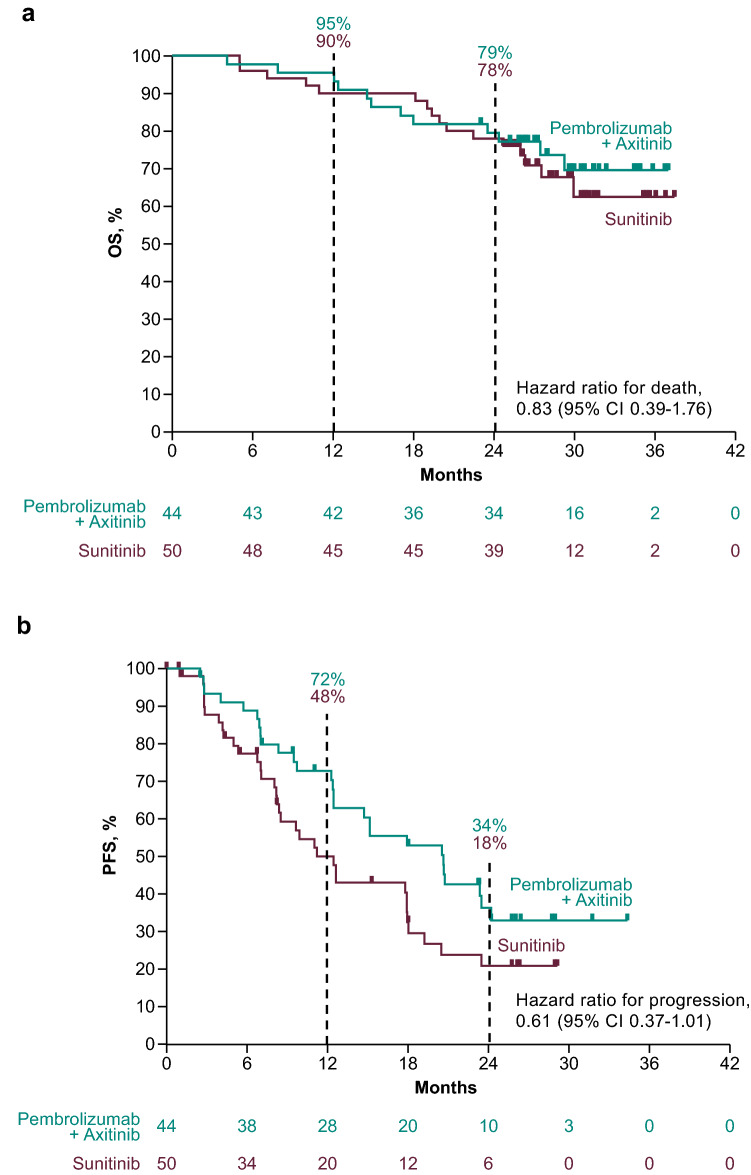
Estimated **a** OS and **b** PFS in the Japanese population (*n* = 94). *CI* confidence interval, *IMDC* International Metastatic Renal Cell Carcinoma Database Consortium, *OS* overall survival, *PFS* progression-free survival

At the data cutoff, 12 (27%) pembrolizumab–axitinib-treated patients and 16 (32%) sunitinib-treated patients in the Japanese subgroup had died, and the median OS was not reached in either treatment group (HR 0.83; 95% CI 0.39–1.76; 12-month rate, 95% vs. 90%; 24-month rate, 79% vs. 78%) (Fig. 2A). Similarly, the median OS was not reached in the global population, but indicated statistically significant improvement for the pembrolizumab–axitinib group at the first interim analysis and persisted at the second interim analysis [10, 12].

At the data cutoff, 27 (61%) pembrolizumab–axitinib-treated patients and 34 (68%) sunitinib-treated patients in the Japanese subgroup had experienced disease progression or died, and the median PFS was 20.6 months (95% CI 12.5–23.5) and 11.3 months (95% CI 8.2–18.0), respectively (HR 0.61; 95% CI 0.37–1.01; 12-month rate, 72% vs. 48%) (Fig. 2B).

**Fig. 3:**
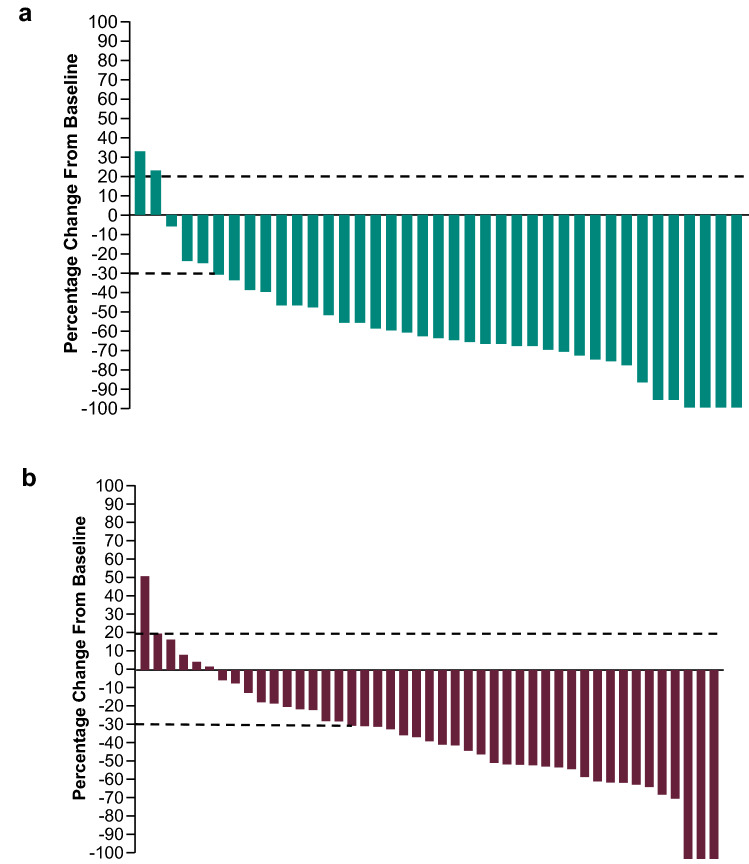
Maximum change from baseline in target lesion size in the **a** pembrolizumab–axitinib group and **b** sunitinib group

**Fig. 4:**
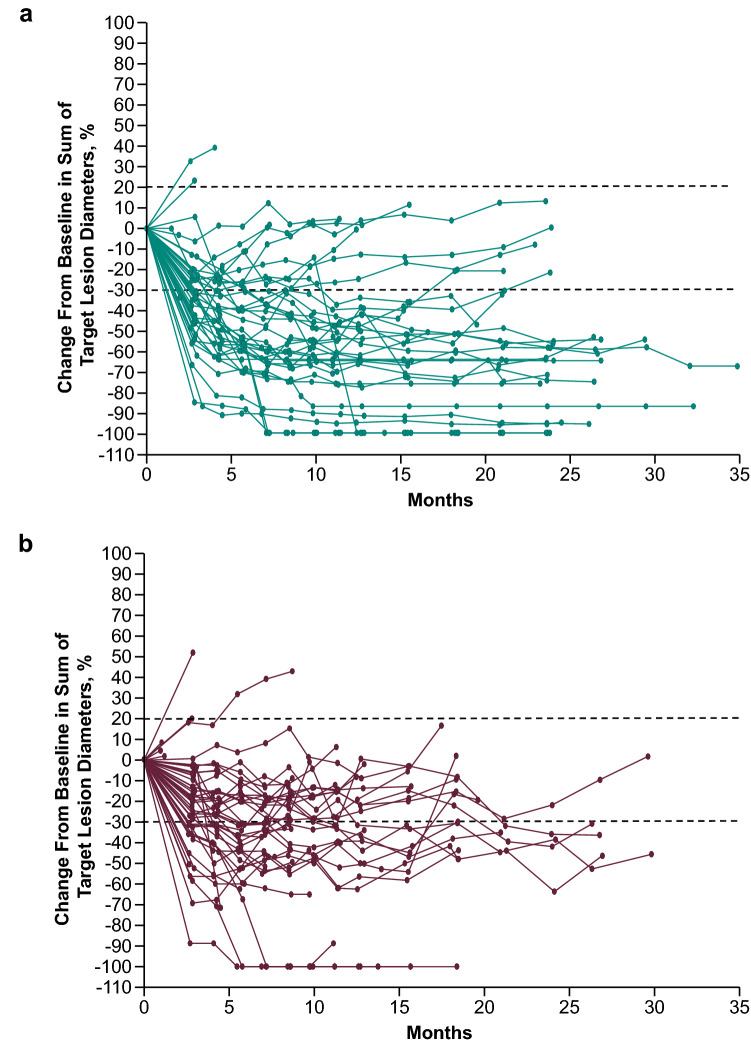
Percentage change from baseline in target lesions in the **a** pembrolizumab–axitinib group and **b** sunitinib group

In the Japanese subgroup, 31/44 patients in the pembrolizumab–axitinib group (ORR 70%; 95% CI 55–83%) and 26/50 patients in the sunitinib group (ORR 52%; 95% CI 37–66%) had a confirmed response (difference, 18%; 95% CI − 2 to 37) (Table 1). A CR was achieved by 6 (14%) patients in the pembrolizumab–axitinib arm and 3 (6%) patients in the sunitinib arm. In the pembrolizumab–axitinib group, a total of 37 of 39 (95%) evaluable patients experienced any reduction in target lesion size; 7 (16%) experienced ≥ 80% reduction (Fig. 3A). In the sunitinib group, 39 of 45 (87%) evaluable patients experienced any reduction in target lesion size; 3 (7%) experienced ≥ 80% reduction (Fig. 3B). Overall, patients in the pembrolizumab–axitinib group experienced greater reductions in tumor size than those in the sunitinib group (Fig. 4). DOR was 16.6 months (4.2 to 29.1+) in pembrolizumab–axitinib-treated patients who achieved a response and 9.9 months (2.8+ to 25.2+) in sunitinib-treated patients who achieved a response (Table 1). The number of patients with response duration of ≥ 12 months as per Kaplan–Meier estimates was 20 (70%) with pembrolizumab–axitinib and 9 (45%) with sunitinib (Table 1).

**Table 1:** Response characteristics in the Japanese population

	Pembrolizumab + Axitinib (*n* = 44)	Sunitinib (*n* = 50)
ORR (CR + PR), % (95% CI)		
Total Japanese population	70.5 (54.8–83.2)	52.0 (37.4–66.3)
Difference	18.5 (− 1.5 to 36.8)	
Best response in Japanese population, *n* (%)		
All patients		
CR	6 (13.6)	3 (6.0)
PR	25 (56.8)	23 (46.0)
SD	9 (20.5)	13 (26.0)
PD	3 (6.8)	6 (12.0)
Not evaluable^a^	0	3 (6.0)
No assessment^b^	1 (2.3)	2 (4.0)
DOR in Japanese population		
Time to response, median (range), months	2.9 (1.9–11.1)	2.8 (2.6–21.0)
DOR, median (range), months	16.6 (4.2 to 29.1+)	9.9 (2.8+ to 25.2+)
Responders at ≥ 12 months , *n* (%)	20 (69.8)	9 (44.5)
Responders at ≥ 24 months, *n* (%)	3 (34.2)	1 (22.6)

*CI* confidence interval; *CR* complete response; *DOR* duration of response; *PD* progressive disease; *PR* partial response; *SD* stable disease

^a^Postbaseline assessments available but not evaluable (i.e., all postbaseline assessments with insufficient data for assessment of response per RECIST v1.1 or CR/PR/SD < 6 weeks from randomization)

^b^No postbaseline assessment available for response evaluation

### Safety

In the as-treated population of the Japanese subgroup, all patients in both treatment groups experienced a treatment-related AE (TRAE); 31 (70%) and 38 (78%) pembrolizumab–axitinib-treated and sunitinib-treated patients experienced a grade 3–4 TRAE, respectively (no grade 5 TRAEs occurred) (Table 2). There were 2 deaths in each group, none of which were due to a TRAE as determined by the investigator (pembrolizumab–axitinib: pleural effusion and pneumonia; sunitinib: urinary tract infection and unknown cause of death). The most commonly occurring TRAEs were palmar–plantar erythrodysesthesia syndrome and hypertension in both arms (Table 2). AEs of interest, which were determined on the basis of a list of terms specified by the sponsor and were considered regardless of whether the investigator determined that they were related to treatment, occurred in 28 (64%) patients in the pembrolizumab–axitinib group and 26 (53%) patients in the sunitinib group; the most common AEs of interest were hypothyroidism and hyperthyroidism in both arms. Grade 3–4 AEs of interest occurred in 8 (18%) pembrolizumab–axitinib-treated patients; none occurred in sunitinib-treated patients (Supplementary Table 3). Among the 44 patients in the pembrolizumab–axitinib group, 9 (20%) patients received high-dose corticosteroids (≥ 40 mg/day prednisone or equivalent for ≥ 1 day) to treat AEs of special interest.

**Table 2:** Treatment-related adverse events (AEs) that occurred in ≥ 20% in either treatment arm

Treatment-related AE	Pembrolizumab-axitinib (*n* = 44)			Sunitinib (*n* = 49)		
	Any grade	Grade 1/2	Grade 3/4	Any grade	Grade 1/2	Grade 3/4
Any	44 (100.0)	13 (29.5)	31 (70.5)	49 (100.0)	11 (22.4)	38 (77.6)
Palmar–plantar erythrodysesthesia	26 (59.1)	24 (54.5)	2 (4.5)	39 (79.6)	35 (71.4)	4 (8.2)
Hypertension	23 (52.3)	17 (38.6)	6 (13.6)	25 (51.0)	16 (32.7)	9 (18.4)
Diarrhea	22 (50.0)	16 (36.4)	6 (13.6)	17 (34.7)	13 (26.5)	4 (8.2)
Proteinuria	18 (40.9)	16 (36.4)	2 (4.5)	14 (28.6)	10 (20.4)	4 (8.2)
Dysphonia	16 (36.4)	16 (36.4)	–	2 (4.1)	2 (4.1)	–
Hypothyroidism	15 (34.1)	15 (34.1)	–	21 (42.9)	21 (42.9)	–
Hepatic function abnormal	13 (29.5)	8 (18.2)	5 (11.4)	5 (10.2)	5 (10.2)	–
Decreased appetite	11 (25.0)	10 (22.7)	1 (2.3)	14 (28.6)	14 (28.6)	–
Blood thyroid-stimulating hormone increased	10 (22.7)	10 (22.7)	–	13 (26.5)	13 (26.5)	–
Fatigue	9 (20.5)	8 (18.2)	1 (2.3)	17 (34.7)	13 (26.5)	4 (8.2)
Stomatitis	9 (20.5)	8 (18.2)	1 (2.3)	17 (34.7)	17 (34.7)	–
Malaise	7 (15.9)	6 (13.6)	1 (2.3)	14 (28.6)	14 (28.6)	–
Dysgeusia	4 (9.1)	4 (9.1)	–	19 (38.8)	19 (38.8)	–
Platelet count decreased	4 (9.1)	3 (6.8)	1 (2.3)	30 (61.2)	13 (26.5)	17 (34.7)
Pyrexia	4 (9.1)	4 (9.1)	–	19 (38.8)	19 (38.8)	–
Neutrophil count decreased	3 (6.8)	2 (4.5)	1 (2.3)	21 (42.9)	8 (16.3)	13 (26.5)
Nausea	2 (4.5)	2 (4.5)	–	10 (20.4)	10 (20.4)	–
Anemia	1 (2.3)	1 (2.3)	–	12 (24.5)	9 (18.4)	3 (6.1)
White blood cell count decreased	0	–	–	22 (44.9)	15 (30.6)	7 (14.3)

Data are *n* (%) and are from the as-treated population

*AE* adverse event

## Discussion

The combination of pembrolizumab–axitinib showed improved OS, PFS, and ORR in the first interim analysis of KEYNOTE-426, and these results persisted in a subsequent analysis [10, 12]. In the Japanese subgroup of KEYNOTE-426, pembrolizumab–axitinib as first-line therapy for mRCC demonstrated efficacy results consistent with those in the overall population, although the sample size was small for this subgroup. Higher rates of AEs have been reported with VEGF/VEGFR therapies in Japanese or Asian patients with RCC [14, 15]; however, the safety and tolerability profiles were similar between this subgroup and the global study population, with balanced rates of TRAEs between the pembrolizumab–axitinib and sunitinib groups. This result was to be expected given that most baseline characteristics were generally similar between the Japanese and total populations and that, as in the global population, most characteristics were balanced between treatment groups within each population. Notable differences were that the Japanese subgroup included older patients, more patients with favorable IMDC risk, and a higher proportion of patients with KPS scale scores of 90/100 versus 70/80 than the global population. Slightly more patients in the Japanese subgroup received subsequent anticancer therapy after discontinuation of study treatment (pembrolizumab–axitinib: 73%; sunitinib: 93%) than in the global population (pembrolizumab–axitinib: 54%; sunitinib: 69%) [12]. Overall, the efficacy and safety results from the KEYNOTE-426 Japanese population are similar to results from the global population, although the Japanese pembrolizumab–axitinib group did not demonstrate OS prolongation. Differences in the distribution of clinical characteristics between the Japanese subgroup and the global pembrolizumab–axitinib population, such as IMDC risk or KPS scores, may have affected the OS results.

Although subset analyses should be viewed with caution given the constraints of small sample sizes, other studies, such as CheckMate 214 and JAVELIN Renal 101, have also reported outcomes in both global and Japanese populations [14, 15]. In CheckMate 214, combination nivolumab–ipilimumab demonstrated a trend in OS over sunitinib for both global and Japanese patients with intermediate or poor IMDC risk [14]. However, PFS rates were not significantly different between the treatment arms for either population. JAVELIN Renal 101 showed that combination avelumab–axitinib significantly improved PFS when compared with sunitinib in the global population, and consistent results were observed in the Japanese patients; OS was not reached for either population [15]. The sunitinib arm of the Japanese population in KEYNOTE-426 demonstrated similar response rates to those in the CheckMate 214 and JAVELIN Renal 101 studies [14, 15]. Across efficacy measures, the results in the Japanese populations in these studies (including KEYNOTE-426) did not differ substantially from those in the global population [12, 14, 15].

In the KEYNOTE-426 Japanese subgroup, the safety profile of pembrolizumab–axitinib was comparable between arms, with manageable AE profiles. Similar rates of TRAEs were observed between the total and Japanese study populations [12]. A slightly higher percentage of patients in the pembrolizumab–axitinib arm of the Japanese population was prescribed high-dose corticosteroids for immune-mediated AEs compared with the same arm of the global population (20% vs. 14%). However, in both analyses, the safety profiles of pembrolizumab, axitinib, and sunitinib were consistent with expectations from prior studies [7, 16, 17]. There were no safety signals in the Japanese subgroup, suggesting pembrolizumab–axitinib combination therapy does not present any additional risk to Japanese patients than was observed in globally derived results [12]. Similarly, both CheckMate 214 and JAVELIN Renal 101 demonstrated comparable safety profiles for the global and Japanese cohorts [14, 15].

Limitations of the analysis presented here include that it was not prespecified, it lacked statistical power due to the small sample size of the Japanese population, and that Japanese nationality was not a stratification factor. In addition, small sample sizes are more likely to be influenced by small imbalances in baseline characteristics, and the lack of stratification in the time-to-event analyses may affect the results.

The results reported here provide long-term safety and efficacy data among the Japanese patient subgroup derived from the extended follow-up analysis of KEYNOTE-426. The results of this long-term post hoc subgroup analysis show that first-line pembrolizumab–axitinib when compared with sunitinib improves ORR and PFS in Japanese patients with advanced clear cell mRCC in a manner similar to that in the global study population. The relative magnitude of the HR for OS in the global population is greater than that observed in the Japanese subgroup. However, the small size of the Japanese subgroup limits interpretation, and the higher use of subsequent therapy in Japanese patients represents an important confounder. Nonetheless, the OS results are consistent between the Japanese and global populations. This analysis verifies the efficacy and safety of pembrolizumab–axitinib for the treatment of mRCC among Japanese patients.

## Supplementary Information

Below is the link to the electronic supplementary material.

Supplementary file1 (PDF 248 KB)

## Data Availability

Merck Sharp & Dohme Corp., a subsidiary of Merck & Co., Inc., Kenilworth, NJ, USA (MSD) is committed to providing qualified scientific researchers access to anonymized data and clinical study reports from the company’s clinical trials for the purpose of conducting legitimate scientific research. MSD is also obligated to protect the rights and privacy of trial participants and, as such, has a procedure in place for evaluating and fulfilling requests for sharing company clinical trial data with qualified external scientific researchers. The MSD data sharing website (http://engagezone.msd.com/ds_documentation.php) outlines the process and requirements for submitting a data request. Applications will be promptly assessed for completeness and policy compliance. Feasible requests will be reviewed by a committee of MSD subject matter experts to assess the scientific validity of the request and the qualifications of the requestors. In line with data privacy legislation, submitters of approved requests must enter into a standard data-sharing agreement with MSD before data access is granted. Data will be made available for request after product approval in the United States and European Union or after product development is discontinued. There are circumstances that may prevent MSD from sharing requested data, including country or region-specific regulations. If the request is declined, it will be communicated to the investigator. Access to genetic or exploratory biomarker data requires a detailed, hypothesis-driven statistical analysis plan that is collaboratively developed by the requestor and MSD subject matter experts; after approval of the statistical analysis plan and execution of a data-sharing agreement, MSD will either perform the proposed analyses and share the results with the requestor or will construct biomarker covariates and add them to a file with clinical data that is uploaded to an analysis portal so that the requestor can perform the proposed analyses.
