# Supplementary material for: Pembrolizumab plus axitinib versus sunitinib in metastatic renal cell carcinoma: outcomes of Japanese patients enrolled in the randomized, phase III, open-label KEYNOTE-426 study
Source: Int J Clin Oncol. 2021 Nov 20;27(1):154–64. doi: 10.1007/s10147-021-02014-7 (PMC8732816; doi:10.1007/s10147-021-02014-7)
Supplement: Supplementary file 1 — Supplementary file1 (PDF 248 KB) [file 10147_2021_2014_MOESM1_ESM.pdf]

SUPPLEMENTAL TABLES AND FIGURES

Pembrolizumab Plus Axitinib versus Sunitinib in Metastatic Renal Cell Carcinoma: Outcomes of Japanese Patients Enrolled in the Randomized, Phase 3, Open-Label KEYNOTE 426 Study

Satoshi Tamada,[1] Chihiro Kondoh,[2] Nobuaki Matsubara,[3] Ryuichi Mizuno,[4] Go Kimura,[5] Satoshi Anai,[6] Yoshihiko Tomita,[7] Masafumi Oyama,[8] Naoya Masumori,[9] Takahiro Kojima,[10] Hiroaki Matsumoto,[11] Mei Chen,[12] Mengran Li,[13]* Kenji Matsuda,[13] Yoshinobu Tanaka,[13] Brian I. Rini,[14] Hirotsugu Uemura[15]

[1]Bell Land General Hospital, Osaka, Japan; [2]Toranomon Hospital, Tokyo, Japan; [3]National Cancer Center Hospital East, Kashiwa, Japan; [4]Keio University Hospital, Tokyo, Japan; [5]Nippon Medical School Hospital, Tokyo, Japan; [6]Nara Medical University Hospital, Kashihara, Japan; [7]Niigata University Medical & Dental Hospital, Niigata, Japan; [8]Saitama Medical University International Medical Center, Saitama Japan, [9]Sapporo Medical University, Sapporo, Japan; [10]University of Tsukuba, Tsukuba, Japan; [11]Yamaguchi University, Yamaguchi, Japan; [12]Merck & Co., Inc., Kenilworth, NJ, USA; [13]MSD K.K., Tokyo, Japan; [14]Cleveland Clinic Taussig Cancer Institute, Cleveland, OH, USA; currently at Vanderbilt-Ingram Cancer Center, Nashville, TN, USA; [15]Kindai University, Osaka, Japan
*At the time the study was conducted

Corresponding author
Satoshi Tamada
Higashiyama 500-3, Naka-ku, Sakai City, Osaka, 599-8247, Japan
satoshitamada@osaka.med.or.jp

Supplementary Table 1 Baseline patient characteristics of the Japanese population

	Japanese Population		Global Population	
	Pembrolizumab + Axitinib (*n* = 44)	Sunitinib (*n* = 50)	Pembrolizumab + Axitinib (*n* = 432)	Sunitinib (*n* = 429)
Male, n (%)	36 (82)	39 (78)	308 (71)	320 (75)
Age, years, median (range/IQR)[a]	66 (43–84)	65 (39–90)	62 (55–68)	61 (53–68)
Age, n (%)				
<65	20 (45)	25 (50)	260 (60)	278 (65)
≥65	24 (55)	25 (50)	172 (40)	151 (35)
Karnofsky Performance Status Scale score, n (%)[b]				
90/100	39 (89)	48 (96)	346 (80)	341 (79)
70/80	5 (11)	2 (4)	85 (20)	88 (21)
IMDC risk category, n (%)				
Favorable	19 (43)	16 (32)	138 (32)	131 (31)
Intermediate/poor	25 (57)	34 (68)	294 (68)	298 (69)
PD-L1 status, n (%)				
CPS ≥1	25 (57)	31 (62)	242 (56)	253 (59)
CPS <1	19 (43)	18 (36)	165 (38)	156 (36)
Missing	0	1 (2)	25 (6)	20 (5)
Sarcomatoid feature, n (%)	7 (16)	5 (10)	51 (12)	54 (13)

CPS combined positive score; *IMDC* International Metastatic Renal Cell Carcinoma Database Consortium; *IQR* interquartile range; *PD-L1* programmed death-ligand 1

[a]Range in the Japanese population; IQR in the global population

[b]One patient had missing information in the global population (pembrolizumab-axitinib group)

Supplementary Table 2 Subsequent therapy after study drug discontinuation in the Japanese population

n (%)	Pembrolizumab + Axitinib (*n* = 44)	Sunitinib (*n* = 50)
Discontinued study treatment	33 (75)	42 (84)
Received any subsequent systemic anticancer therapy after discontinuation[a]	24 (73)	39 (93)
Any VEGF/VEGFR inhibitor[b]	18 (55)	26 (62)
Nivolumab[c]	4 (12)	28 (67)
Other type[d]	6 (18)	4 (10)
1 subsequent line	24 (73)	39 (93)
2 subsequent lines	6 (18)	19 (45)
≥3 subsequent lines	1 (3)	6 (14)

PD-L1 programmed death ligand 1; *VEGF* vascular endothelial growth factor; *VEGFR* vascular endothelial growth factor receptor

[a]Percentages are calculated with 33 discontinuations as the denominator

[b]Axitinib, cabozantinib, pazopanib, sorafenib, or sunitinib

[c]Only PD-L1 inhibitor used as subsequent therapy

[d]Everolimus, interferon (unspecified), investigational drug (unspecified), ipilimumab, temsirolimus.

Supplementary Table 3 Adverse events (AEs) of interest

	Pembrolizumab-axitinib (*n* = 44)			Sunitinib (*n* = 49)		
AEs of interest	Any grade	Grade 1/2	Grade 3/4	Any grade	Grade 1/2	Grade 3/4
Any	28 (63.6)	20 (45.5)	8 (18.2)	26 (53.1)	26 (53.1)	0
Hypothyroidism	15 (34.1)	15 (34.1)	0	21 (42.9)	21 (42.9)	0
Hyperthyroidism	7 (15.9)	7 (15.9)	0	2 (4.1)	2 (4.1)	0
Adrenal insufficiency	5 (11.4)	5 (11.4)	0	0	0	0
Colitis	5 (11.4)	2 (4.5)	3 (6.8)	0	0	0
Pneumonitis	3 (6.8)	2 (4.5)	1 (2.3)	1 (2.0)	1 (2.0)	0
Thyroiditis	3 (6.8)	3 (6.8)	0	2 (4.1)	2 (4.1)	0
Myasthenic syndrome	2 (4.5)	2 (4.5)	0	0	0	0
Myositis	2 (4.5)	2 (4.5)	0	0	0	0
Hepatitis	1 (2.3)	0	1 (2.3)	0	0	0
Hypophysitis	1 (2.3)	0	1 (2.3)	0	0	0
Infusion reactions	1 (2.3)	1 (2.3)	0	1 (2.0)	1 (2.0)	0
Myocarditis	1 (2.3)	0	1 (2.3)	0	0	0
Nephritis	1 (2.3)	1 (2.3)	0	1 (2.0)	1 (2.0)	0
Severe skin reactions	1 (2.3)	0	1 (2.3)	1 (2.0)	1 (2.0)	0
Uveitis	1 (2.3)	1 (2.3)	0	0	0	0

Data are *n* (%) and are from the as-treated population.

Supplementary Figure 1. Patient disposition of the Japanese population. *AE* adverse event; *CR* complete response; *PD* progressive disease

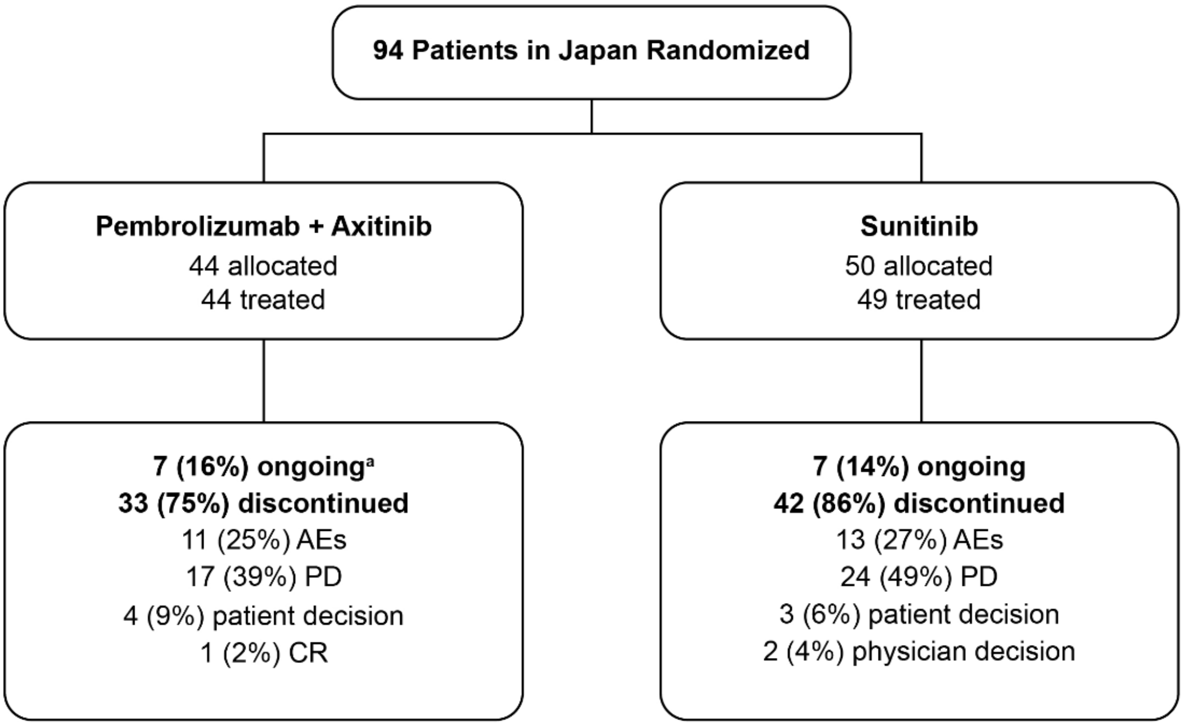

[a]Four patients completed 2 full years of pembrolizumab.
